# TIME SPENT IN SEDENTARY BEHAVIOR DOMAINS AND PHYSICAL FUNCTION IN U.S. OLDER ADULTS

**DOI:** 10.1093/geroni/igz038.602

**Published:** 2019-11-08

**Authors:** Nancy M Gell, Erin D Bouldin, Dori Rosenberg

**Affiliations:** 1 University of Vermont, Burlington, Vermont, United States; 2 Appalachian State University, Boone, North Carolina, United States; 3 Kaiser Permanente Washington Health Research Institute, Seattle, Washington, United States

## Abstract

Previous studies have reported associations of sedentary time with worse health outcomes in older adults. Yet, little is known about the relationships between the contexts of sedentary time and health outcomes. The purpose of this study was to examine associations of physical function with time spent in a variety of sedentary behavior domains. We analyzed data from the 2016 National Health and Aging Trends Study, a nationally representative sample of community-dwelling Medicare beneficiaries. Estimated time being sedentary by domains (e.g., TV watching, computer use, resting/napping, eating, transportation, socializing, sitting and doing hobbies) were collected from a subset of the sample population (N=2157). The Short Physical Performance Battery (SPPB) measured physical function. Linear regression models were conducted adjusting for sociodemographics, health conditions, pain, and dementia. More time watching TV and resting/napping was significantly associated total SPPB scores (p < 0.01). In adjusted models, lower SPPB scores were significantly associated with more time/day spent sitting and watching television or resting (ɮ =-0.16 hours; 95% Confidence Interval (CI): -.024, -0.08 for TV watching and ɮ =-0.63 hours; 95% CI: -0.80, -0.46 for resting). Average time in computer use, eating, transportation, hobbies, or social activities did not differ by physical function level. Associations between physical function and sedentary time vary by the context. Social or engaging sedentary activities do not appear to be associated with physical function limitations in the same way as passive sedentary domains like television viewing and resting. Context should be considered in evaluating relationships of sedentary time with health outcomes.

